# Supplementary material for: Single prolonged stress blocks sleep homeostasis and pre-trauma sleep deprivation does not exacerbate the severity of trauma-induced fear-associated memory impairments
Source: PLoS One. 2021 Jan 6;16(1):e0243743. doi: 10.1371/journal.pone.0243743 (PMC7787370; doi:10.1371/journal.pone.0243743)
Supplement: Attachment — Submitted filename: Response to Reviewers.docx [file pone.0243743.s002.docx]

Reviewer #1: Authors hypothesized that sleep deprivation (SD) prior to trauma exposure may increase the severity of a PTSD-like phenotype in rats exposed to single prolonged stress (SPS), a rodent model of PTSD and investigated "the contribution of pre-trauma sleep loss to subsequent trauma-dependent fear-associated memory impairmen". They also announced that that they identified a unique time frame where trauma exposure and sleep may interact and identifies this window of time as a potential therapeutic treatment window for staving off the negative consequences of trauma exposure. However, the results in this manuscript might not give these conclussion.

1. Because the REMs and NREMs in SD and SD+SPS groups showed no difference, it chould not be conclused that "Treatment-specific sleep phenotypes are shown for the SPS and SPS+SD animals".

**We agree with the reviewer’s observation, the sentence as written was not precise. Therefore, we altered this sentence to highlight the main finding that the homeostatic response to sleep loss is different between SPS and SPS+SD animals (see lines 56-60).**

2. What are the practical clinical implications of poor sleep prior to trauma for PTSD?

**Sleep loss can occur as the result of acute deprivation (as in our study here) or as a series of prolonged sleep reductions that occur as a result of social and societal pressures to extend waking due to work/personal life requirements. The former seems insufficient to cause exaggerated performance impairment, while the latter has been extensively examined in the context of the development of mood/cognitive impairments even in the absence of trauma. We now include a more thorough discussion on the impact of sleep loss on trauma exposure and cite relevant literature (see lines 443-457).**

Reviewer #2: • In the entire article, the units should be abbreviated according to the SI system. (e.g.: 15 minutes: 15 min, 2 hours: 2h, 21-24 degree C: 21-24 °C).

**We made these suggested abbreviation changes.**

• It was reported that male rats were used in the study. The reason for using especially male rats in the experiments should be stated in the animal's section.

**We amended the animal section to read the following: Male, Long Evans rats were used for all experiments to eliminate the known impact of estrous cycle hormones on sleep and behavior [19, 20, 21] (see lines 114-115).**

• Adding an experimental design drawing that schematizes the experimental protocols used by the researchers makes the article both easier to understand and more visual.

**At the reviewer’s request, we have added an experimental schematic as Fig 1 (referenced in the “Experimental timeline” subheading of the methods section).**

• It would be more appropriate to write 72.99 ±7.9 % instead of M = 72.99 ±7.9 percent in statistical expressions.

**We made these changes as suggested.**

Reviewer #3: In the present manuscript, the authors investigated the effects of sleep deprivation on PTSD-like symptoms following trauma. The authors hypothesized that sleep deprivation (SD) prior to trauma exposure may increase the severity of a PTSD-like phenotype in rats exposed to single prolonged stress (SPS). However, they found that sleep loss prior to trauma exposure did not further exaggerate the PTSD-like phenotype as there was no difference in freezing between the SPS and the SPS+SD group on the fear recall day. The findings are interesting, but the manuscript have major fundamental problems as mentioned below:

1. The authors claim that fear-associated memory processing was impaired after SPS was incorrect since there was an increase in the freezing percent in SPS and SPS-SD groups during the extinction phase which suggest that animals retained their memory about the trauma associated with the cue.

**Indeed, increased freezing on the extinction day was found in SPS exposed animals compared to non-SPS-exposed animals. We interpret this increase in extinction day freezing as an aberrant behavioral response that indicates that SPS resulted in cued extinction deficits. If SPS exposed animals were not impaired, they would have shown reduced freezing, and therefore, increased freezing is seen as a deficit in this case. We now discuss this aberrant behavioral response in further detail in the discussion section (see lines 384-397).**

2. Another problem with the results was that they did not find any effect of extinction in the SPS group. On the fear recall day, animals in the SPS group displayed significant increase in the freezing.

**The reviewer has highlighted a main finding from this work. SPS exposed rats show “extinction retention deficits”. This means that trauma exposed animals still freeze on the recall day and that the extinction trial was insufficient to return the animals to baseline. We clarified the distinction between extinction retention deficits on the recall day and increased freezing on the conditioning and extinction day in the discussion section (lines 384-408).**

3. In the SD group, they did not find any increased freezing during fear conditioning which they did not discuss in the discussion. But interestingly, their freezing percent increased during fear extinction. Again, no explanation in the discussion.

**We made significant changes to the discussion section to address the reviewer’s concern about cued extinction deficits and have included a more thoughtful explanation about the role of “time of day” in the acquisition of these fear-associated memory tasks.**

4. In addition, there was difference in the degree of freedom in Figure 1. In fear condition, it was 19, in fear extinction, it was 119 whereas in fear recall, it was 39. I do not understand why authors have used different number of animals on each of these three days.

**The number of animals is unchanged throughout these experiments. The degrees of freedom were different between these three tests because they each contained different number of data points. Freezing behavior is measured in 1 min bins and we report the averaged percent time across the 5 data sampling points on the fear conditioning day, 30 data sampling points for the extinction day, and 10 data sampling points on the recall day. This is the source of the discrepancy in degrees of freedom on these 3 measures.**
